# The Threat Called *Candida haemulonii* Species Complex in Rio de Janeiro State, Brazil: Focus on Antifungal Resistance and Virulence Attributes

**DOI:** 10.3390/jof8060574

**Published:** 2022-05-27

**Authors:** Lívia S. Ramos, Maria Helena G. Figueiredo-Carvalho, Laura N. Silva, Nahyara L. M. Siqueira, Joice C. Lima, Samuel S. Oliveira, Rodrigo Almeida-Paes, Rosely M. Zancopé-Oliveira, Fabio S. Azevedo, Adriana L. P. Ferreira, Marta H. Branquinha, André L. S. Santos

**Affiliations:** 1 Laboratório de Estudos Avançados de Microrganismos Emergentes e Resistentes (LEAMER), Departamento de Microbiologia Geral, Instituto de Microbiologia Paulo de Góes (IMPG), Universidade Federal do Rio de Janeiro (UFRJ), Rio de Janeiro 21941-901, RJ, Brazil; liviaramos2@yahoo.com.br (L.S.R.); lauransilva@gmail.com (L.N.S.); nayh_182@hotmail.com (N.L.M.S.); joicebiotec@gmail.com (J.C.L.); samueloliveira.bio@outlook.com (S.S.O.); mbranquinha@micro.ufrj.br (M.H.B.); 2 Laboratório de Micologia, Instituto Nacional de Infectologia Evandro Chagas, Fundação Oswaldo Cruz (FIOCRUZ), Rio de Janeiro 21040-360, RJ, Brazil; maria.helena@ini.fiocruz.br (M.H.G.F.-C.); rodrigo.paes@ini.fiocruz.br (R.A.-P.); rosely.zancope@ini.fiocruz.br (R.M.Z.-O.); 3 Rede Micologia RJ—Fundação de Amparo à Pesquisa do Estado do Rio de Janeiro (FAPERJ), Rio de Janeiro 21941-901, RJ, Brazil; 4 Laboratório Diagnóstico da América SA (DASA), Duque de Caxias, Rio de Janeiro 25085-007, RJ, Brazil; fsazevedo@dasa.com.br (F.S.A.); adriana.pires@dasa.com.br (A.L.P.F.); 5 Programa de Pós-Graduação em Bioquímica (PPGBq), Instituto de Química, Universidade Federal do Rio de Janeiro (UFRJ), Rio de Janeiro 21941-909, RJ, Brazil

**Keywords:** *Candida haemulonii* complex, antifungal resistance, biofilm formation, hydrolytic enzymes, hemolysins, siderophores

## Abstract

Although considered rare, the emergent *Candida haemulonii* species complex, formed by *C. haemulonii sensu stricto* (*Ch*), *C. duobushaemulonii* (*Cd*) and *C. haemulonii* var. *vulnera* (*Chv*), is highlighted due to its profile of increased resistance to the available antifungal drugs. In the present work, 25 clinical isolates, recovered from human infections during 2011–2020 and biochemically identified by automated system as *C. haemulonii*, were initially assessed by molecular methods (amplification and sequencing of *ITS1-5.8S-ITS2* gene) for precise species identification. Subsequently, the antifungal susceptibility of planktonic cells, biofilm formation and susceptibility of biofilms to antifungal drugs and the secretion of key molecules, such as hydrolytic enzymes, hemolysins and siderophores, were evaluated by classical methodologies. Our results revealed that 7 (28%) isolates were molecularly identified as *Ch*, 7 (28%) as *Chv* and 11 (44%) as *Cd*. Sixteen (64%) fungal isolates were recovered from blood. Regarding the antifungal susceptibility test, the planktonic cells were resistant to (i) fluconazole (100% of *Ch* and Chv, and 72.7% of *Cd* isolates), itraconazole and voriconazole (85.7% of *Ch* and Chv, and 72.7% of *Cd* isolates); (ii) no breakpoints were defined for posaconazole, but high MICs were observed for 85.7% of *Ch* and *Chv*, and 72.7% of *Cd* isolates; (iii) all isolates were resistant to amphotericin B; and (iv) all isolates were susceptible to echinocandins (except for one isolate of *Cd*) and to flucytosine (except for two isolates of *Cd*). Biofilm is a well-known virulence and resistant structure in *Candida* species, including the *C. haemulonii* complex. Herein, we showed that all isolates were able to form viable biofilms over a polystyrene surface. Moreover, the mature biofilms formed by the *C. haemulonii* species complex presented a higher antifungal-resistant profile than their planktonic counterparts. Secreted molecules associated with virulence were also detected in our fungal collection: 100% of the isolates yielded aspartic proteases, hemolysins and siderophores as well as phospholipase (92%), esterase (80%), phytase (80%), and caseinase (76%) activities. Our results reinforce the multidrug resistance profile of the *C. haemulonii* species complex, including Brazilian clinical isolates, as well as their ability to produce important virulence attributes such as biofilms and different classes of hydrolytic enzymes, hemolysins and siderophores, which typically present a strain-dependent profile.

## 1. Introduction

The *Candida haemulonii* complex is formed by the uncommon species *C. haemulonii* *sensu stricto*, *C. duobushaemulonii* and *C. haemulonii* var. *vulnera*, and it is phylogenetically related to *C. auris*, *C. pseudohaemulonii* and *C. vulturna*. They belong to the Metschnikowiaceae clade and require molecular methods for correct identification; the amplification and sequencing of the *ITS* gene being considered the gold standard [1]. The isolation frequency of the *C. haemulonii* species complex has been increasing worldwide over the last 10 years and risk factors are mainly associated with previous therapy with antimicrobial agents, including antifungals, while the major underlying diseases are malignant tumors, organ transplants, diabetes mellitus and vascular diseases [2].

Although infections caused by the species comprising the *C. haemulonii* complex are considered rare, their multidrug resistance profile to the current available antifungal drugs is alarming, particularly to amphotericin B and azoles, being frequently related to clinical failure especially in invasive infections [1,3]. Primary evidence suggests that amphotericin B resistance in the *C. haemulonii* species complex was considered multifactorial, involving lower amounts of ergosterol in the plasma membrane, dysfunction of mitochondria, increased resistance to oxidative stressors and the upregulation of antioxidant machinery [4]. Additionally, drug efflux pumps and multiple mutations in *ERG11* genes seem to mediate azole resistance in this emerging fungal complex [5].

The *C. haemulonii* species complex can cause diseases with varied clinical manifestations, from superficial to invasive infections. Several reports have elicited a range of cellular characteristics and virulence attributes that could help them to overcome the hostile environment of the human body and cause illness. In this sense, the ability to adhere to abiotic substrates, including different catheter types and, consequently, biofilm formation is of great importance in hospital settings once it facilitates the access to bloodstream and dissemination to internal organs [6]. Additionally, the production of different classes of biologically active secreted molecules such as hydrolytic enzymes (e.g., proteases, phospholipases, esterases, and phytases) as well as hemolysins and siderophores allows them to hydrolyze key components of tissues and cells as well as to evade from both cellular and humoral host immune responses [7,8]. Recently, the description of phenotypic switching in *C. haemulonii*, i.e., the ability to switch between different morphological types, reinforces the capacity of rapid adaptation to changes in host environment, contributing to its survival in different host niches [9].

In the present work, we aimed to study 25 clinical isolates, previously identified by an automated method as *C. haemulonii*, recovered from Brazilian hospitals (Rio de Janeiro State) over a period of 10 years (2011–2020). To do this, firstly, the fungal isolates were precisely identified by using molecular methods (amplification and sequencing of *ITS1-5.8S-ITS2* gene). Subsequently, the antifungal susceptibility profiles of both planktonic- and biofilm-forming cells were evaluated. Finally, the production of important virulence attributes associated with *Candida* infection, such as biofilm formation and the secretion of biologically active molecules (e.g., hydrolytic enzymes, hemolysins and siderophores), were also investigated in our fungal collection.

## 2. Materials and Methods

### 2.1. Microorganisms and Growth Conditions

**Figure 1:**
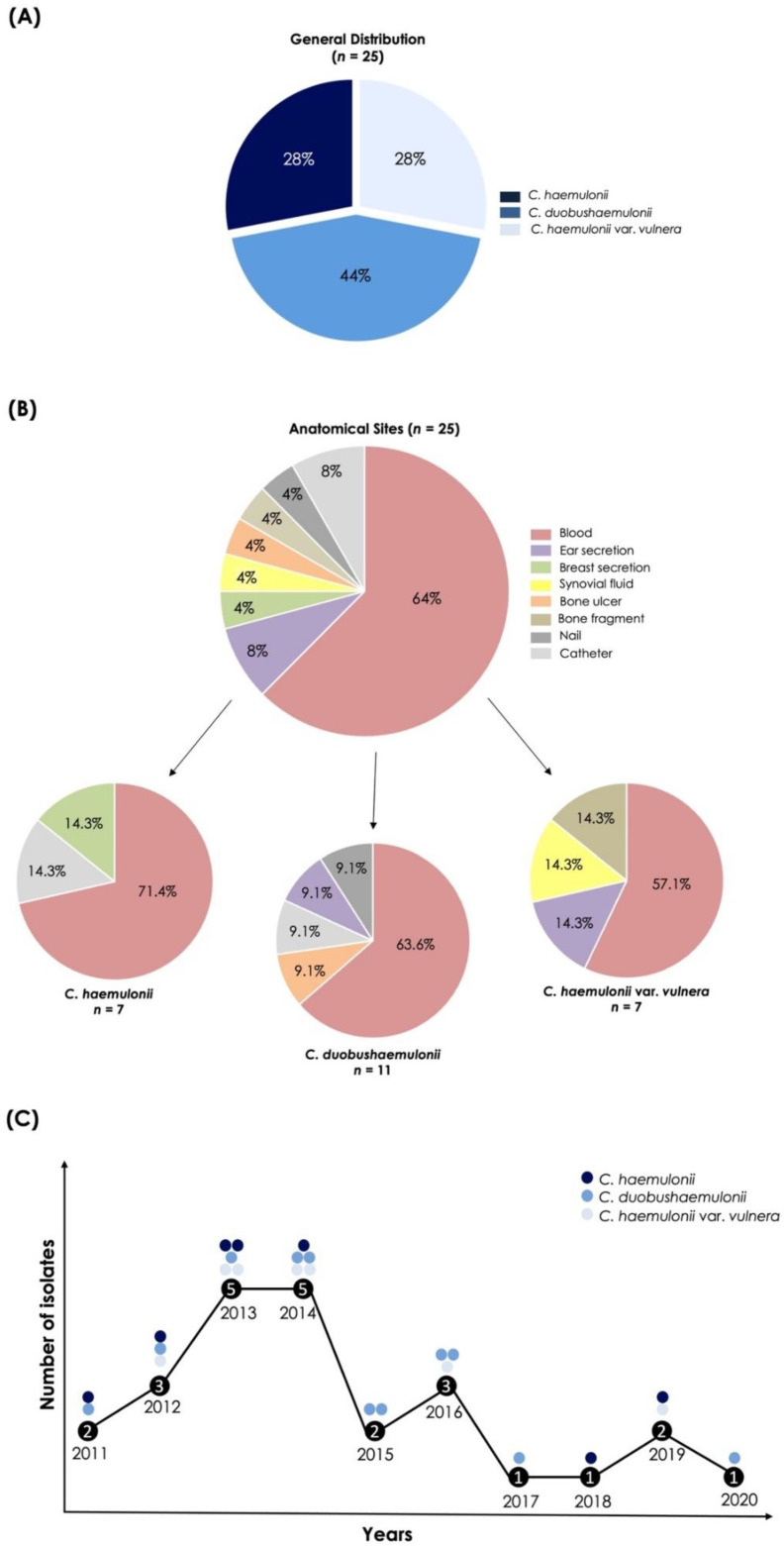
Distribution of species comprising the *C. haemulonii* complex obtained from Brazilian hospitals (Rio de Janeiro State). (**A**) General species distribution considering the total number of clinical isolates (*n* = 25). (**B**) Percentage of clinical isolates obtained from each anatomical site considering the total number of fungal isolates, followed by the anatomical sites’ distribution per species forming the *C. haemulonii* complex. (**C**) Timeline comprising the distribution of species of the *C. haemulonii* complex used in the present work recovered per year. The numbers inside the black dots represent the number of total isolates obtained in the corresponding year.

Twenty-five clinical isolates, previously identified by biochemical approaches as *C. haemulonii*, recovered from patients in Brazilian hospitals (Rio de Janeiro State) between 2011 and 2020 (Figure 1), were studied. The isolates were obtained from several clinical specimens, such as blood (*n* = 16), ear secretion (*n* = 2), breast secretion (*n* = 1), synovial fluid (*n* = 1), bone ulcer (*n* = 1), bone fragment (*n* = 1), nail (*n* = 1), and catheter (*n* = 2). Prior to the experiments, these clinical fungal isolates were recovered from storage (–20 °C) and grown in Sabouraud dextrose agar (Sigma-Aldrich, St. Louis, MO, USA) and CHROMagar^TM^ Candida medium (Becton, Dickinson and Company, Franklin Lakes, NJ, USA), both at 37 °C for 48 h, in order to evaluate their viability and purity, respectively. The phenotypic confirmation of the species after storage was achieved by biochemical analyses with the Vitek^®^ 2 system (bioMérieux, Marcy l’Etoile, France) using the YST card according to the manufacturer’s instructions. For the subsequent experiments, fungal cells were cultured into Sabouraud dextrose medium (37 °C/48 h/200 rpm).

### 2.2. Molecular Identification

Yeast cells obtained from pure colonies were recovered from Sabouraud dextrose agar and used for DNA extraction with the Gentra^®^ Puregene^®^ Yeast and G+ Bacteria Kit (Qiagen, Germantown, MD, USA). The clinical isolates were identified by sequencing the *ITS1-5.8S-ITS2* gene region of the rDNA as previously described [1], using the following primers: ITS1 (5′–TCCGTAGGTGAACCTGCGG–3′) and ITS4 (5′–TCCTCCGCTTATTGATATGC–3′). Sequences were edited using Sequencher^TM^ version 4.9 and compared by BLAST with sequences available from the NCBI/GenBank database.

### 2.3. Antifungal Susceptibility Assay

Susceptibility testing was performed according to the standardized broth microdilution technique described by Clinical & Laboratory Standards Institute (CLSI) in document M27-A3 [10]. Antifungal drugs tested were amphotericin B, fluconazole, itraconazole, voriconazole, posaconazole, caspofungin, micafungin, anidulafungin, flucytosine, and terbinafine (Sigma-Aldrich, St. Louis, MO, USA). The minimal inhibitory concentrations of the drugs on planktonic yeast cells (MICs) were determined according to the CLSI M27S3 protocol: susceptible (S) ≤ 8 mg/L, susceptible-dose dependent (S-DD) 16–32 mg/L and resistant (R) ≥ 64 for fluconazole; S ≤ 0.125 mg/L, S-DD 0.25–0.5 mg/L and R ≥ 1 mg/L for itraconazole; S ≤ 1 mg/L, S-DD 2 mg/L and R ≥ 4 mg/L for voriconazole; S ≤ 2 mg/L and nonsusceptible (NS) > 2 mg/L for caspofungin, micafungin and anidulafungin; S ≤ 4 mg/L, intermediate (I) 8–16 mg/L and R ≥ 32 mg/L for flucytosine [11]. No breakpoints have been defined for posaconazole, terbinafine and amphotericin B by the CLSI protocol, but in general clinical isolates of *Candida* spp. with MIC values > 1 mg/L are considered resistant to amphotericin B [12]. In parallel, we also used the tentative breakpoints suggested for *C. auris* by the Centers for Disease Control and Prevention (CDC, Atlanta, GE, USA), since the *C. haemulonii* species complex are phylogenetic related to *C. auris* as follows: R ≥ 32 for fluconazole; R ≥ 2 for amphotericin B and caspofungin; R ≥ 4 for micafungin and anidulafungin.

### 2.4. Biofilm Formation

Fungal cell suspensions in Sabouraud broth (200 µL containing 10^6^ cells) were transferred into each well of a flat-bottom 96-well polystyrene microtiter plate and then incubated without agitation at 37 °C for 48 h. Medium-only blanks were also set up in parallel. Subsequently, the supernatant fluids were carefully removed and the wells were washed three times with PBS to remove non-adherent fungal cells. Biomass quantification was assessed as described by Peeters and co-workers [13]. First, biofilms were fixed with 200 μL of 99% methanol for 15 min. The supernatants were then discarded. Microtiter plates were air-dried for 5 min and then 200 μL of 0.4% crystal violet solution (stock solution diluted in PBS; Sigma-Aldrich, St. Louis, MO, USA) was added to each well and the plates were incubated at room temperature for 20 min. The wells were washed once with PBS to remove excess stain and the biomass in each well was then decolorized with 200 μL of 33% acetic acid for 5 min. One hundred microliters of the acetic acid solution was transferred to a new 96-well plate and the absorbance was measured at 590 nm using a microplate reader (SpectraMax M3; Molecular Devices, Sunnyvale, CA, USA). The metabolic activity of the biofilm was determined using a colorimetric assay able to measure the metabolic reduction of 2,3-bis (2-methoxy-4-nitro-5-sulfophenyl)-5-[(phenylamino) carbonyl]-2H-tetrazolium hydroxide (XTT; Sigma-Aldrich, St. Louis, MO, USA) to a water-soluble brown formazan product [14]. The XTT/menadione solution was prepared by dissolving 2 mg XTT in 10 mL of pre-warmed PBS, which was supplemented with 100 μL of a stock solution of menadione (0.4 mM in acetone). The XTT/menadione solution (200 μL) was added to the plate wells and incubated at 37 °C for 3 h in the dark. Afterwards, 100 μL of the supernatant from each well was transferred to a new microplate and the colorimetric changes were quantified using a microplate reader at 492 nm (SpectraMax M3; Molecular Devices, San Jose, CA, USA).

### 2.5. Antifungal Susceptibility of Biofilm-Forming Cells

In this assay, we selected one antifungal agent from each class for which the clinical isolates were susceptible in the antifungal susceptibility assay. The clinical isolates were incubated at 37 °C for 48 h to allow biofilm formation, as described above. Afterwards, the supernatant was carefully removed, and the biofilm was washed once with sterile PBS. Then, an aliquot of 200 μL of RPMI-1640 buffered with MOPS and supplemented with the antifungals prepared according to the CLSI M27-A3 [10] protocol was added. The plates were incubated at 37 °C for an additional 48 h. Finally, XTT reduction assay was performed to evaluate biofilm viability. The minimal biofilm eradication concentrations (MBECs) were determined as the lowest concentrations of the antifungal agents that were able to reduce at least 50% of cell viability compared with the drug-free growth control well [15,16].

### 2.6. Production of Biologically Active Extracellular Molecules

The production of extracellular molecules by fungal cells was carried out in agar plate assays as described previously by Price et al. [17]. Briefly, the aspartic protease activity was determined using 1.17% yeast carbon base (YCB) medium supplemented with 1% bovine serum albumin (BSA) according to Rüchel et al. [18]. Caseinolytic activity was assessed using Sabouraud dextrose agar containing 0.4% casein as previously described by Ziccardi et al. [19]. The determination of phospholipase activity was performed using egg yolk agar plate (1 M NaCl, 5 mM CaCl_2_ and 2% sterile egg yolk emulsion, pH 7.0) as previously described by Price et al. [17]. The esterase production was assayed using the Tween agar plate (peptone, 1 g; NaCl, 0.5 g; CaCl_2_, 0.01 g; agar, 1.5%; Tween, 0.1%; pH 7.0) according to Aktas et al. [20]. Phytase activity was evaluated using the calcium phytate agar (glucose, 10 g; (NH_4_)_2_SO_4_, 0.5 g; KCl, 0.2 g; MgSO_4_·7H_2_O, 0.1 g; calcium phytate, 2 g; yeast extract, 0.5 g; MnSO_4_, 0.005 g; FeSO_4_, 0.005 g, pH 7.0) according to Tsang [21]. Hemolysin production was evaluated by adding 7 mL of fresh sheep blood to 100 mL of Sabouraud dextrose agar supplemented with 3% glucose (final concentration, wt/vol) [22]. Siderophore production was determined using blue indicator dye, chrome azurol S (CAS); 60.5 mg CAS were dissolved in 50 mL water and mixed with 10 mL iron (III) solution (1 mM FeCl_3_·6H_2_O, 10 mM HCl); under stirring this solution was slowly added to 72.9 mg hexadecyltrimethylammonium (HDTMA) dissolved in 40 mL water at 50 °C and then autoclaved; the final mixture of 100 mL was added to 900 mL of autoclaved Sabouraud dextrose medium [23]. To determine the production of these extracellular molecules, aliquots (10 µL) of 48 h-old cultured fungal cells (10^7^ cells/mL) were spotted on the surface of each agar medium and incubated at 37 °C for up to 7 days. The colony diameter (a) and the diameter of the colony plus the hydrolysis/precipitation zone (b) were measured by a digital paquimeter and the production of each molecule was expressed as *Pz* value (a/b) as previously described [17]. The *Pz* value was scored into four categories: *Pz* of 1.0 indicated no production; *Pz* between 0.999 and 0.700 indicated weak producers; *Pz* between 0.699 and 0.400 corresponded to good producers; and *Pz* lower than 0.399 mean excellent producers [17].

### 2.7. Statistics

All experiments were performed in triplicate, in three independent experimental sets. The results were analyzed statistically by the Analysis of Variance One-Way ANOVA (comparisons between three or more groups). All analyses were performed using the program GraphPad Prism8. In all analyses, *p* values of 0.05 or less were considered statistically significant.

## 3. Results and Discussion

### 3.1. Origin and Proper Identification of the C. haemulonii Clinical Isolates

The clinical isolates used in the present work were previously identified as *C. haemulonii* by an automated method. To confirm the identity of the isolates, we carried out some distinct methodologies. First, the clinical isolates were incubated in CHROMagar^TM^ Candida medium for 48 h at 37 °C and all of them developed colonies with light to dark violet color. Several reports demonstrated that species belonging to the *C. haemulonii* complex usually develop this pigmentation in this particular chromogenic medium [3], but many other *Candida* species also develop similar colors in this medium, which demands additional methods to correctly identify these emerging fungal species.

The biochemical identification performed by the Vitek^®^ 2 YST system, which evaluates both carbohydrate assimilation and metabolic enzymatic profiles, identified five isolates as *C. haemulonii* (probability of identity ranging from 90 to 99%), eight as *C. duobushaemulonii* (probability of identity ranging from 90 to 97%) and twelve isolates were uncertainly identified as possible two or three different species (four as *C. haemulonii*/*Kodamaea ohmeri*, six as *C. haemulonii*/*C. haemulonii* var. *vulnera*, one as *C. auris*/*C. duobushaemulonii* and one as *C. haemulonii*/*Cryptococcus neoformans*/*C. duobushaemulonii*) (Table 1). The Vitek 2^®^ system software was updated in 2017 to the version 8.01, which includes *C. auris*, *C. duobushaemulonii*, *C. haemulonii* var. *vulnera* and *Cryptococcus gattii* [24]; before that, *C. auris*, *C. duobushaemulonii* and *C. haemulonii* var. *vulnera* were identified as *C. haemulonii*. However, based on literature data and our results, phenotypic methods cannot correctly identify the members of the *C. haemulonii* species complex and the closely related species, with the use of molecular approaches being necessary. Based on these statements, the sequencing of the *ITS1-5.8S-ITS2* gene after PCR was considered as the gold standard to precisely identify our clinical fungal isolates. In this sense, seven isolates (28%) were identified as *C. haemulonii*, seven (28%) as *C. haemulonii* var. *vulnera* and eleven (44%) as *C. duobushaemulonii* (Figure 1A). Relevantly, biochemical and molecular identification methods were in accordance only in 40% of our studied fungal collection. However, when analyzing the species individually, we observed that 72.7% (8/11) of *C. duobushaemulonii* isolates were in accordance in both methods of identification employed, while for *C. haemulonii* isolates this percentage was 25.6% (2/7). On the other hand, none of *C. haemulonii* var. *vulnera* isolates exhibited accordance between both methods.

Analyzing the isolation sites of our fungal collection (Figure 1B), the majority (16/25; 64%) of the *C. haemulonii* species complex were recovered from blood as follows: 71.4% of *C. haemulonii* isolates, 63.6% of *C. duobushaemulonii* and 57.1% of *C. haemulonii* var. *vulnera*. *C. haemulonii* was obtained from only two more sites (breast secretion and catheter) and *C. haemulonii* var. *vulnera* from three more different sites (ear secretion, synovial fluid and bone fragment), while *C. duobushaemulonii* isolates were obtained from four different anatomical sites: ear secretion, bone ulcer, nail and catheter (Figure 1B). The species distribution of the *C. haemulonii* complex over the 10 years (2011–2020) of research is also shown, revealing that at least one fungal strain was isolated in each considered year (Figure 1C).

### 3.2. Antifungal Susceptibility Assay

Concerning the antifungal susceptibility tests and considering the breakpoints suggested in the document CLSI M27S3 for *Candida* spp. [11], since no specific breakpoints were designated until now to the species belonging to the *C. haemulonii* complex, most of the clinical isolates were resistant to the azole members tested. In this sense, all isolates of *C. haemulonii* and *C. haemulonii* var. *vulnera* were resistant to fluconazole (MICs ranging from 64 to >64 mg/L) as well as 72.7% of the *C. duobushaemulonii* isolates (MICs ranging from 8 to >64 mg/L), while 85.7% of *C. haemulonii* and *C. haemulonii* var. *vulnera* isolates and 72.7% of *C. duobushaemulonii* isolates exhibited resistance to itraconazole and voriconazole (MICs ranging from 0.125 to >16 mg/L for both itraconazole and voriconazole) (Table 2, Table 3 and Table 4). MICs for posaconazole ranged from 0.06 to >16 mg/L, but no breakpoints have been defined for this azole. On the other hand, all clinical isolates were susceptible to the three tested echinocandins (caspofungin, micafungin and anidulafungin), with the exception of one isolate of *C. duobushaemulonii* which was resistant to caspofungin (Table 2, Table 3 and Table 4). Only two isolates of *C. duobushaemulonii* exhibited resistance to flucytosine, while all the remaining 23 isolates belonging to the *C. haemulonii* complex were susceptible to this antifungal agent (MICs ranging from 0.25 to >64 mg/L) (Table 2, Table 3 and Table 4). Although CLSI has no defined breakpoints to amphotericin B, *Candida* isolates with MICs >1 mg/L are usually considered resistant. Based on this premise, the 25 clinical isolates of *C. haemulonii* species complex were considered to be resistant to amphotericin B (MICs ranging from 2 to >16 mg/L). Finally, all isolates exhibited high MICs values to terbinafine (ranging from 1 to >16 mg/L), but breakpoints to this antifungal agent have not been defined yet (Table 2, Table 3 and Table 4).

Since document CLSI M27S3 is not recent, we also compared our results with the tentative breakpoints suggested by CDC for *C. auris*, which is a close phylogenetic species. In this sense, differences in the resistance classification were observed only in fluconazole for *C. duobushaemulonii* isolates, which presented 90.9% of resistant isolates according to CDC breakpoints instead of 72.7% according to CLSI (Table 4). The breakpoints for amphotericin B, micafungin and anidulafungin were the same in both documents, and one concentration below for caspofungin (but it did not influence the classification of the clinical fungal isolates studied herein). No breakpoints have been suggested by CDC for the other antifungals used in the present work.

Our results are in agreement with those reported in the literature for this fungal complex, with a commonly observed increased resistance to fluconazole [1,25,26,27,28,29,30,31,32,33], itraconazole, voriconazole, posaconazole and amphotericin B [1,3,27,32,33,34]. In general, the *C. haemulonii* complex exhibits in vitro susceptibility to echinocandins [3,29,33,35,36,37], although there are reports of resistance to this antifungal class [1,38]. Little information about its susceptibility profile to flucytosine and terbinafine is available in the literature but, like us, Lima et al. [39] reported low MICs (<0.5 mg/L) for flucytosine against the three members of the *C. haemulonii* complex and Pagani et al. [34] reported high MICs (>16 mg/L) for terbinafine against environmental isolates of *C. haemulonii*.

### 3.3. Biofilm Formation and Antifungal Susceptibility of Biofilm-Forming Cells

**Figure 2:**
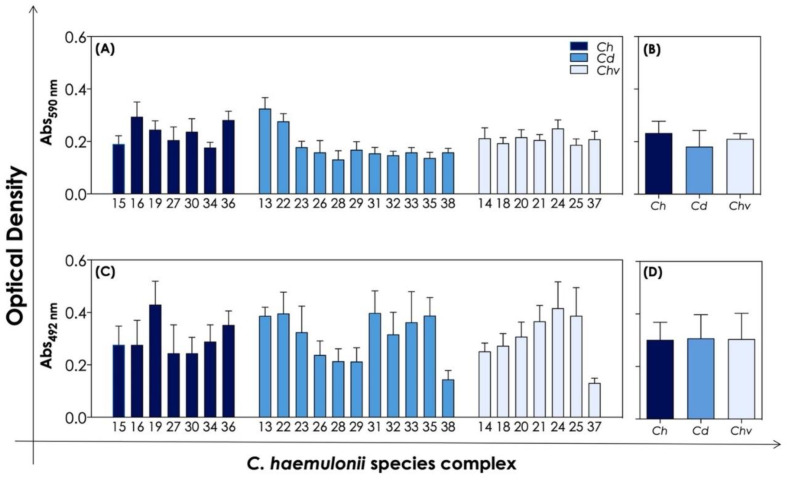
Biofilm formation by *C. haemulonii* species complex on a polystyrene surface. Biofilms were formed for 48 h at 37 °C and then processed to detect the fungal biomass (590 nm) and cell viability (492 nm). The results are expressed as absorbance (ABS) values per clinical isolate studied (**A**,**C**) and mean per fungal species (**B**,**D**). The results represent means ± standard deviation of three independent experiments. The numbers on the *X*-axis of the graph represent each of the 25 isolates of the *C. haemulonii* complex used, in which *Ch* means *C. haemulonii*, *Cd* means *C. duobushaemulonii* and *Chv* means *C. haemulonii* var. *vulnera*.

Biofilm formation is a well-known virulence attribute with recognized importance for the infection process of various bacteria and fungi [37,38,39,40]. *C. albicans* biofilm formation has been extensively studied over the years and much progress has been made in the study of biofilm formation by some non-*albicans Candida* species, particularly *C. tropicalis*, *C. glabrata*, *C. krusei* and *C. parapsilosis*. Indeed, the ability of the *C. haemulonii* species complex to form biofilm has also been demonstrated by some authors [6,40,41]. In this sense, the biofilm formation by the clinical isolates of the *C. haemulonii* species complex used herein was initially evaluated by means of a crystal violet assay to measure the biomass of mature biofilm (Figure 2A,B). The results showed that our clinical isolates were able to produce biofilm biomass in a quite similar way (Figure 2A), exhibiting means of absorbance of 0.233 ± 0.044 for *C. haemulonii* isolates, 0.181 ± 0.061 for *C. duobushaemulonii*, and 0.210 ± 0.020 for *C. haemulonii* var. *vulnera* (Figure 2B). No significant differences (*p* > 0.05; one-way ANOVA, Tukey’s multiple comparison test) were observed in the mean values considering the biofilm formation capacity among the three species forming the *C. haemulonii* complex (Figure 2B). However, some variations in the ability to produce biofilm biomass were observed when comparing the strains individually; for instance, strains 13 and 22 of *C. duobushaemulonii* produced led to two times more biomass than the remaining strains belonging to this species (Figure 2A). Similar observations can be extrapolated from the analysis of the biofilm viability (Figure 2C). Viability was assessed by the metabolic activity of living cells forming the mature biofilm of *C. haemulonii* species complex that were able to convert XTT to formazan (Figure 2C), presenting means of absorbance of 0.302 ± 0.067 for *C. haemulonii* isolates, 0.307 ± 0.090 for *C. duobushaemulonii*, and 0.305 ± 0.097 for *C. haemulonii* var. *vulnera* (Figure 2D). All these results are in accordance with those observed by our group while testing biofilm formation by 12 clinical isolates of the *C. haemulonii* complex [6,41]. Additionally, Cendejas-Bueno et al. [1] also observed similar results regarding biofilm biomass formation by isolates of the *C. haemulonii* complex. Lima et al. [42] suggested that high biofilm production by isolates of *C. duobushaemulonii* and *C. haemulonii* var. *vulnera* can impact the pathogenicity of these microorganisms on in vivo *Caenorhabditis elegans* infection model.

Comparing the biofilm formation capability (both biomass and viability parameters) of our fungal collection recovered from blood and those obtained from other anatomical sites, no significant differences were observed for none of the analyzed biofilm parameters (*p* > 0.05; unpaired Student *t* test).

The biofilm lifestyle confers several advantageous features to *Candida* cells that contribute to infection progression, for example, resistance against host immune responses and other environmental stress situations, as well as increased resistance to antifungal agents in comparison with their planktonic counterparts [43]. For these reasons, biofilm-related infections pose a challenge to successful treatment, resulting in the microbial persistence and development of chronic diseases [44]. In order to evaluate the antifungal susceptibility profile of biofilm-forming cells, we selected anidulafungin (an echinocandin) and flucytosine (an antimetabolite), to which the vast majority of the isolates were susceptible, for further experimentation. In general, the MBEC of the isolates tested were at least two times higher than the values observed for planktonic cells, except for three isolates for which no differences were observed. Anidulafungin was more efficient than flucytosine at reducing the viability of biofilm-forming cells of the clinical isolates of the *C. haemulonii* complex. In this sense, 42.9% of the biofilms formed by *C. haemulonii* isolates were resistant to anidulafungin, while 71.4% were resistant/intermediate to flucytosine; regarding *C. duobushaemulonii* biofilms, the percentage of resistance was the same for both antifungals (27.3%). On the other hand, the biofilms formed by all isolates of *C. haemulonii* var. *vulnera* were susceptible to anidulafungin, whilst 57.1% were resistant/intermediate to flucytosine (Table 5).

Previously, our group reported that biofilms formed by clinical isolates of the *C. haemulonii* complex were susceptible to caspofungin and micafungin [45], demonstrating that echinocandins can still be considered a treatment option to infections caused by these yeasts. Additionally, echinocandins has also been reported to be active against biofilms formed by *C. albicans*, *C. parapsilosis* and *C. glabrata*, but not against *C. tropicalis*, evidencing that the differences in action can be related to species-specific characteristics [46,47]. Contrarily, Romera et al. [16] reported that biofilms formed by *C. auris*, a species that also belongs to the *C. haemulonii* clade, were resistant to different antifungal classes used, exhibiting MBECs up to 250 times higher than planktonic MICs for amphotericin B and up to 512 times higher for echinocandins (anidulafungin and caspofungin) and azoles (fluconazole and voriconazole), reinforcing the idea that planktonic cells are mostly susceptible to these antifungal agents. This is in accordance with various works that have shown the augmented resistance profile of *Candida* spp. biofilms, such as *C. albicans*, *C. tropicalis*, *C. parapsilosis* and others [44]. Such a phenomenon can be explained by some reasons, including the (i) high density of cells forming the biofilm, which present different growth rates, metabolic activities and sometimes varied morphologies; (ii) the presence of the extracellular matrix, which protects the fungal cells within the biofilm, working as a filter/barrier that limits the penetration of antifungal drugs; (iii) differential expression of drug targets; and (iv) the common activation of drug efflux pumps [44].

### 3.4. Production of Extracellular Biologically Active Molecules Associated with Fungal Virulence

**Figure 3:**
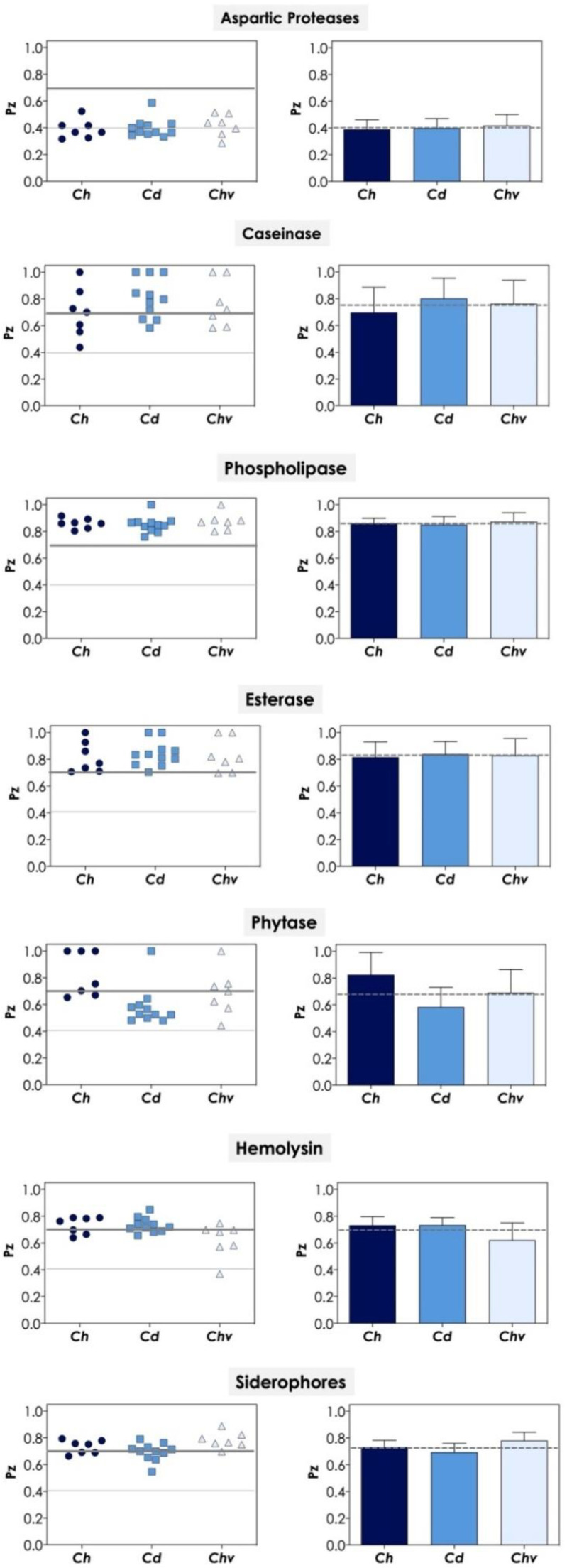
Hydrolytic enzymes produced by *C. haemulonii* species complex. The distribution of aspartic protease, caseinase, phospholipase, esterase, phytase, hemolysin and siderophores production in each clinical isolate was shown (**left side**). Note that the gray lines divide the graph according to the intensity of enzymatic activity as follows: below the thin gray line are found the excellent producing fungal isolates, between the gray lines are found the good producing isolates and above the gray thick line are found the weak producing strains. In parallel, the mean ± standard deviation regarding the aspartic protease, caseinase, phospholipase, esterase, phytase, hemolysin and siderophores production in each species forming the *C. haemulonii* complex was plotted (**right side**). Note that the dotted line represents the overall average for the production of each enzyme in all studied strains (*n* = 25). *Ch*, *C. haemulonii*; *Cd*, *C. duobushaemulonii*; *Chv*, *C. haemulonii* var. *vulnera*.

It is well known that *Candida* cells can secrete into the extracellular environment several classes of molecules with direct/indirect impacts on fungal virulence, such as hydrolytic enzymes, hemolysins and siderophores, which facilitate their colonization, the establishment of the infectious process, survival and persistence within the host [48]. Due to their relevance, we decided to investigate the production of biologically active extracellular molecules, including aspartic proteases, caseinases, phospholipases, esterases, phytases, hemolysins and siderophores in the *C. haemulonii* species complex by means of detection on agar plates, as depicted in Figure 3 and described below.

The secretion of aspartic-type proteases (collectively called as Saps) is considered one of the most important virulence factors involved in the pathogenicity of *Candida* spp. and their expression is induced by the in vitro growth of yeast cells in chemically defined media supplemented with large proteins (e.g., albumin and hemoglobin) as the only nitrogenous source [49]. Our results revealed that all the 25 clinical isolates of *C. haemulonii* complex studied herein were able to produce aspartic proteases, exhibiting moderate to strong enzymatic activities as judged by the *Pz* values, which ranged from 0.286 to 0.586. The production of aspartic proteases by the *C. haemulonii* species complex has been reported in clinical isolates from Brazil [7,50] and in isolates obtained from soft corals on Brazilian reefs [34]; in both works, all isolates tested were able to produce this enzyme class. The expression of aspartic-type proteases in *Candida* spp. is encoded by *SAP* gene families that differ among *Candida* species. For example, *C. albicans SAP* family is composed of 10 different genes, whereas *C. dubliniensis*, *C. tropicalis* and *C. parapsilosis* possess, respectively, eight, four and three different genes [51]. Until now, no *SAP* gene family has been described in the *C. haemulonii* complex, but the genome proteins database of *C. haemulonii* and *C. duobushaemulonii* possess, respectively, six and eight proteins with a Sap-like conserved domain, and most of them exhibit predictive molecular masses similar to Saps1-3 of *C. albicans* [50]. Indeed, our group demonstrated that *C. albicans* anti-Saps1-3 antibodies were able to recognize Sap-like proteins on the cell surface of isolates comprising the *C. haemulonii* complex [50].

It is recognized that *Candida* cells cultured in a complex medium (e.g., brain heart infusion, Sabouraud, casein medium, etc.) can secrete proteolytic enzymes other than aspartic-type proteases [52]. In this sense, the secretion of serine proteases has already been reported in clinical isolates of *C. albicans* and some non-*albicans Candida* species, including *C. dubliniensis*, *C. tropicalis*, *C. guilliermondii* [53,54] and also in the *C. haemulonii* species complex [8], all of them capable of cleaving a broad spectrum of proteins, including important key host components such as serum proteins (e.g., hemoglobin, immunoglobulin and albumin) [8,53,54]. Herein, we showed that moderate to weak caseinolytic activity was detected in 85.7% (6/7) of isolates of *C. haemulonii*, 72.7% (8/11) of *C. duobushaemulonii* and 71.4% (5/7) of *C. haemulonii* var. *vulnera*, with *Pz* ranging from 0.436 to 0.843. In our previous work, on the other hand, all 12 clinical isolates of the *C. haemulonii* complex tested were positive for caseinolytic activity [7], while Pagani et al. [34] demonstrated that 75% of *C. haemulonii* isolates from soft corals tested were strong producers of caseinase and 25% exhibited no activity. Additionally, our group reported that clinical isolates of the *C. haemulonii* complex were able to secrete serine proteases able to hydrolyze casein, later demonstrating that the extracellular serine proteases belong to the S1 family, exhibiting chymotrypsin-, trypsin- and elastase-like activities [8,55].

Phospholipases are enzymes able to cause the disruption of the plasma membrane of host cells by the hydrolysis of one or more ester linkages in glycerophospholipids, releasing free fatty acids [56]. Although phospholipases possess target phospholipid substrates, their classification is based on the specific ester bond they target [56]. In this sense, *C. albicans* owns phospholipases A, B, C and D, but until now only few members of phospholipase B are known to be secreted to the environment [57]. In vivo experiments demonstrated that the absence of phospholipase B resulted in the attenuation of virulence of *C. albicans* in a murine model of disseminated candidiasis [58]. Under our experimental conditions, weak phospholipase activity was detected in all *C. haemulonii* isolates (7/7, 100%), 90.9% (10/11) of *C. duobushaemulonii* isolates and 85.7% (6/7) of *C. haemulonii* var. *vulnera* isolates, with *Pz* ranging from 0.760 to 0.916. Similar results were observed by Pagani et al. [34], in which a weak activity of this lipolytic enzyme was detected in all environmental isolates of *C. haemulonii* tested, and by our group after testing 12 human clinical isolates of the *C. haemulonii* complex [7]. Additionally, phospholipase activity has been detected in some non-*albicans Candida* species, such as *C. parapsilosis*, *C. tropicalis*, *C. krusei*, *C. guilliermondi* and others [59,60].

Esterases, another class of lipolytic enzymes, can hydrolyze the ester bonds of mono-, di- and triacylglycerols into alcohol and acid, acting better on soluble substrates [48]. Esterases are involved in lipid digestion for nutritional purposes, and during the infection they participate in the adhesion process and invasion of epithelial cells [57]. Our results showed that esterase activity was quite similar to the phospholipase activity, with 85.7% (6/7), 81.8% (9/11) and 71.4% (5/7) of isolates exhibiting weak activity for *C. haemulonii*, *C. duobushaemulonii* and *C. haemulonii* var. *vulnera*, respectively (*Pz* ranging from 0.696 to 0.926). Our group previously described moderate to weak esterase activity in clinical isolates of the *C. haemulonii* complex [7], while Pagani et al. [34] observed strong esterase activity in almost all (7/8) environmental isolates tested. Besides *C. albicans* and the *C. haemulonii* species complex, esterase activity has been detected in various non-*albicans Candida* species, such as *C. parapsilosis*, *C. tropicalis*, *C. krusei*, *C. glabrata*, and *C. dubliniensis* [19,61,62,63].

Phytate is the main phosphorus store in plants, representing more than 60% of the total phosphorus content. Consequently, phytate is found in abundance in animal and human diets and thus in their gastrointestinal tracts. Phytase catalyzes the hydrolysis of phytic acid, releasing organic phosphate and inositol, thus allowing microorganisms to acquire phosphate and inositol from the host. Both products are considered essential nutrients for all living cells. Phytase activity seems to contribute to survival and proliferation, for example, within the gastrointestinal tract, where nutrients are scarce [21]. Mutations in the synthesis and transport of inositol, essential components of glycosylphosphatidylinositol anchors, resulted in non-viable, avirulent *C. albicans* cells [64]. Herein, a moderate to weak activity of phytase was observed in 57.1% (4/7) of isolates of *C. haemulonii*, 90.9% (10/11) of *C. duobushaemulonii* and 85.7% (6/7) of *C. haemulonii* var. *vulnera*, with *Pz* ranging from 0.443 to 0.756. On the other hand, we observed in our previous work that all clinical isolates of the *C. haemulonii* complex were able to secrete phytase with mainly moderate activity [7]. Phytase activity has been reported in many *Candida* species, such as *C. albicans*, *C. glabrata*, *C. guilliermondii*, *C. tropicalis*, *C. parapsilosis* and others [21]. Indeed, Tsang [21] reported different phytase activities in distinct *Candida* species and, also, among isolates from the same species; in this sense, a higher percentage of positive isolates were from *C. albicans*, *C. glabrata* and *C. guilliermondii*, while the higher enzymatic activity was detected in isolates of *C. krusei*, *C. kefyr* and *C. albicans* [21].

Hemolysins are enzymes able to promote the lysis of red blood cells through plasma membrane damage, allowing iron acquisition from hemoglobin, which supports the growth and development of *Candida* cells and, also, facilitates their dissemination through the bloodstream and the establishment of the infectious process [57]. Moderate to weak hemolytic activity was detected in all isolates of our fungal collection, with the exception of one isolate of *C. haemulonii* var. *vulnera* that exhibited excellent hemolytic activity; *Pz* values ranged from 0.370 to 0.850. In our previous work with clinical isolates of the *C. haemulonii* complex, we also reported hemolytic activity in all isolates tested, but most of them exhibited excellent to moderate activity [7]. The production of hemolysins seems to be very prevalent both in *C. albicans* and non-*albicans Candida* species, such as *C. glabrata*, *C. tropicalis*, *C. krusei* and *C. guilliermondii* [22,57]. In this sense, Chin et al. [65] reported that *C. albicans* isolates from a Malaysian hospital exhibited higher hemolytic activity than non-*albicans Candida* isolates. On the other hand, Riceto et al. [66] did not observe significant differences in hemolytic activity between *C. albicans* and non-*albicans Candida* species. Interestingly, non-*albicans Candida* isolates (*C. dubliniensis*, *C. krusei* and *C. glabrata*) obtained from the oral cavity of diabetic individuals exhibited hemolytic activitiessignificantly higher than healthy individuals, while *C. albicans* isolates presented no significant differences between the two groups studied [62].

Siderophores are ferric iron-specific chelators with low molecular weight secreted by most fungi to solubilize the iron present in the environment. Iron is an essential micronutrient for all living organisms and once its acquisition is a key step during the infection process, siderophores are considered classically virulence factors [67]. All the clinical isolates tested produced siderophores, exhibiting mainly weak activity, with *Pz* ranging from 0.546 to 0.888.

No significant differences were observed among the mean *Pz* value of each species of the *C. haemulonii* complex for each extracellular molecule investigated in the present study, which means that the three members of the complex produce similar amounts of each extracellular molecule (*p* > 0.05; one-way ANOVA; Tukey’s multiple comparison test). Additionally, no significant difference was observed when comparing the secretion of each extracellular molecule from isolates obtained from blood and those obtained from other anatomical sites (*p* > 0.05; unpaired Student *t* test).

## 4. Conclusions

The emergence of multidrug-resistant fungi in recent decades has been viewed with concern by scientists and healthy authorities in many countries all over the world, especially due to the limited arsenal of antifungal options to treat such infections. Besides the drug resistance, these fungi possess a range of strategies to overcome the hostile environment of the human body, culminating in infection. The *C. haemulonii* species complex is part of this worrisome group of emergent microorganisms and, collectively, our results reinforce the multidrug resistance profile of this fungal complex, including Brazilian clinical isolates, as well as their ability to produce important virulence attributes such as biofilms and different classes of biologically relevant extracellular molecules, which contribute in different stages of the infection process.

## Figures and Tables

**Table 1:** Biochemical and molecular identification of the *C. haemulonii* species complex used in the present work.

Code	Source of Isolates	Isolation Year	Identification by Vitek^®^ 2 YST System (Probability of Identity)	Molecular Identification (*ITS1-5.8S-ITS2* Gene)	GenBank Accession Number
LIP*Ch*13	Blood	2012	*C. haemulonii* (90%)	*C. duobushaemulonii*	OM575048
LIP*Ch*14	Blood	2012	*C. haemulonii* (97%)	*C. haemulonii* var. *vulnera*	OM575049
LIP*Ch*15	Blood	2012	*C. haemulonii/Kodamaea ohmeri*	*C. haemulonii*	OM575050
LIP*Ch*16	Catheter tip	2013	*C. haemulonii* (97%)	*C. haemulonii*	OM575051
LIP*Ch*18	Blood	2014	*C. haemulonii* (99%)	*C. haemulonii* var. *vulnera*	OM575052
LIP*Ch*19	Blood	2011	*C. haemulonii/Kodamaea ohmeri*	*C. haemulonii*	OM575053
LIP*Ch*20	Blood	2013	*C. haemulonii/Kodamaea ohmeri*	*C. haemulonii* var. *vulnera*	OM575054
LIP*Ch*21	Ear secretion	2016	*C. haemulonii/Kodamaea ohmeri*	*C. haemulonii* var. *vulnera*	OM575055
LIP*Ch*22	Blood	2015	*C. auris/C. duobushaemulonii*	*C. duobushaemulonii*	OM575056
LIP*Ch*23	Blood	2011	*C. duobushaemulonii* (93%)	*C. duobushaemulonii*	OM575057
LIP*Ch*24	Blood	2014	*C. haemulonii/C. haemulonii* var. *vulnera*	*C. haemulonii* var. *vulnera*	OM575058
LIP*Ch*25	Synovial fluid	2013	*C. haemulonii/C. haemulonii* var. *vulnera*	*C. haemulonii* var. *vulnera*	OM575059
LIP*Ch*26	Blood	2016	*C. duobushaemulonii* (92%)	*C. duobushaemulonii*	OM575060
LIP*Ch*27	Blood	2018	*C. haemulonii/C. haemulonii* var. *vulnera*	*C. haemulonii*	OM575061
LIP*Ch*28	Bone ulcer	2017	*C. duobushaemulonii* (92%)	*C. duobushaemulonii*	OM575062
LIP*Ch*29	Catheter tip	2016	*C. duobushaemulonii* (90%)	*C. duobushaemulonii*	OM575063
LIP*Ch*30	Breast secretion	2014	*C. haemulonii* (95%)	*C. haemulonii*	OM575064
LIP*Ch*31	Ear secretion	2014	*C. duobushaemulonii* (90%)	*C. duobushaemulonii*	OM575065
LIP*Ch*32	Blood	2015	*C. haemulonii/C. neoformans/C. duobushaemulonii*	*C. duobushaemulonii*	OM575066
LIP*Ch*33	Blood	2013	*C. duobushaemulonii* (90%)	*C. duobushaemulonii*	OM575067
LIP*Ch*34	Blood	2013	*C. haemulonii/C. haemulonii* var. *vulnera*	*C. haemulonii*	OM575068
LIP*Ch*35	Blood	2014	*C. duobushaemulonii* (90%)	*C. duobushaemulonii*	OM575069
LIP*Ch*36	Blood	2019	*C. haemulonii/C. haemulonii* var. *vulnera*	*C. haemulonii*	OM575070
LIP*Ch*37	Bone fragment	2019	*C. haemulonii/C. haemulonii* var. *vulnera*	*C. haemulonii* var. *vulnera*	OM575071
LIP*Ch*38	Nail	2020	*C. duobushaemulonii* (97%)	*C. duobushaemulonii*	OM575072

**Table 2:** MIC values of antifungal agents against each clinical isolate of *C. haemulonii* species complex studied in the present work.

Clinical Isolates		MIC (mg/L) ^a^								
	FLC ^b^	ITC	VRC	PSC	AMB	TRB	5-FC	CSF	MCF	ANF
***Ch***										
LIP*Ch*15	>64	>16	>16	>16	>16	8	0.5	1	0.125	0.25
LIP*Ch*16	>64	>16	>16	>16	4	8	0.5	1	0.06	0.125
LIP*Ch*19	>64	>16	>16	>16	2	16	0.25	1	0.125	0.25
LIP*Ch*27	>64	>16	>16	>16	2	>16	0.5	1	0.125	0.125
LIP*Ch*30	>64	>16	>16	>16	8	>16	0.5	1	0.125	0.125
LIP*Ch*34	>64	>16	>16	>16	4	>16	0.125	1	0.06	0.06
LIP*Ch*36	64	0.25	1	0.25	16	>16	0.25	0.25	<0.015	0.125
***Cd***										
LIP*Ch*13	8	0.125	0.12	0.25	2	1	>64	>8	0.125	2
LIP*Ch*22	>64	8	>16	>16	16	16	0.5	0.5	0.06	0.125
LIP*Ch*23	64	8	16	16	>16	>16	0.25	0.5	0.125	0.06
LIP*Ch*26	>64	>16	>16	>16	>16	>16	1	0.5	0.06	0.06
LIP*Ch*28	>64	>16	8	>16	4	>16	0.25	0.125	0.06	0.06
LIP*Ch*29	64	>16	16	>16	>16	>16	0.25	0.5	0.125	0.06
LIP*Ch*31	>64	0.5	0.5	0.125	4	>16	1	0.125	0.06	0.06
LIP*Ch*32	>64	>16	>16	>16	>16	>16	0.125	0.5	0.125	0.06
LIP*Ch*33	64	>16	>16	>16	4	>16	0.125	0.25	0.125	0.06
LIP*Ch*35	32	0.5	0.5	0.06	4	8	0.5	0.25	0.125	0.06
LIP*Ch*38	32	0.5	>16	>16	>16	>16	>64	0.25	0.06	0.125
***Chv***										
LIP*Ch*14	>64	>16	>16	>16	2	8	0.25	0.25	0.06	0.125
LIP*Ch*18	>64	>16	>16	>16	4	8	0.25	1	0.125	0.125
LIP*Ch*20	>64	>16	>16	>16	8	16	0.25	1	0.125	0.25
LIP*Ch*21	>64	>16	>16	>16	4	8	0.25	1	0.125	0.06
LIP*Ch*24	64	0.25	0.25	0.125	16	>16	0.25	1	0.125	0.06
LIP*Ch*25	>64	>16	>16	>16	4	>16	0.25	0.5	0.06	0.06
LIP*Ch*37	>64	>16	>16	>16	8	>16	<0.125	0.5	0.06	0.06

^a^ MIC, minimal inhibitory concentration; *Ch*, *C. haemulonii*; *Cd*, *C. duobushaemulonii*; *Chv*, *C. haemulonii* var. *vulnera*; ^b^ FLC, fluconazole; ITC, itraconazole; VRC, voriconazole; PSC, posaconazole; AMB, amphotericin B; TRB, terbinafine; 5-FC, flucytosine; CSF, caspofungin; MCF, micafungin; ANF, anidulafungin.

**Table 3:** MIC ranges, MIC_50_ and MIC_90_ values of the *C. haemulonii* species complex to classical antifungal agents.

Antifungals	*Ch* (*n* = 7)			*Cd* (*n* = 11)			*Chv* (*n* = 7)		
	MIC Range	MIC_50_	MIC_90_	MIC Range	MIC_50_	MIC_90_	MIC Range	MIC_50_	MIC_90_
AMB	2–>16	4	16	2–>16	16	>16	2–16	4	8
FLC	64–>64	>64	>64	8–>64	64	>64	64–>64	>64	>64
ITC	0.25–>16	>16	>16	0.125–>16	8	>16	0.25–>16	>16	>16
VRC	1–>16	>16	>16	0.125–>16	16	>16	0.25–>16	>16	>16
PSC	0.25–>16	>16	>16	0.06–>16	>16	>16	0.125–>16	>16	>16
CSF	0.25–1	1	1	0.125–>8	0.5	0.5	0.25–1	1	1
MCF	<0.015–0.125	0.125	0.125	0.06–0.125	0.125	0.125	0.06–0.125	0.125	0.125
ANF	0.06–0.25	0.125	0.25	0.06–2	0.06	0.125	0.06–0.25	0.06	0.125
5-FC	0.125–0.5	0.5	0.5	0.125–>64	0.5	>64	<0.125–0.25	0.25	0.25
TRB	8–>16	>16	>16	1–>64	>16	>16	8–>16	16	>16

MIC, minimal inhibitory concentration. All MIC values are shown in mg/L. *Ch*, *C. haemulonii*; *Cd*, *C. duobushaemulonii*; *Chv*, *C. haemulonii* var. *vulnera*; FLC, fluconazole; ITC, itraconazole; VRC, voriconazole; PSC, posaconazole; AMB, amphotericin B; TRB, terbinafine; 5-FC, flucytosine; CSF, caspofungin; MCF, micafungin; ANF, anidulafungin; MIC_50_, MIC at which 50% of growth was inhibited; MIC_90_, MIC at which 90% of growth was inhibited.

**Table 4:** Percentage of resistant strains of *C. haemulonii* species complex studied herein based on both CLSI and CDC breakpoints.

Species			% of Resistant Strains					
	FLC CLSI/CDC	ITC CLSI/CDC	VRC CLSI/CDC	AMB CLSI/CDC	5-FC CLSI/CDC	CSF CLSI/CDC	MCF CLSI/CDC	ANF CLSI/CDC
***Ch***	100%/100%	85.7%/ND	85.7%/ND	100%/100%	0%/ND	0%/0%	0%/0%	0%/0%
***Cd***	72.7%/90.9%	72.7%/ND	72.7%/ND	100%/100%	18.2%/ND	9.1%/9.1%	0%/0%	0%/0%
***Chv***	100%/100%	85.7%/ND	85.7%/ND	100%/100%	0%/ND	0%/0%	0%/0%	0%/0%

*Ch*, *C. haemulonii*; *Cd*, *C. duobushaemulonii*; *Chv*, *C. haemulonii* var. *vulnera*; ND, non-determined, since no breakpoint is available until now. FLC, fluconazole; ITC, itraconazole; VRC, voriconazole; AMB, amphotericin B; 5-FC, flucytosine; CSF, caspofungin; MCF, micafungin; ANF, anidulafungin. Breakpoints for posaconazole and terbinafine have been not defined by either CLSI or CDC.

**Table 5:** Comparison of the susceptibility profile between planktonic- and biofilm-forming cells of the *C. haemulonii* complex isolates against flucytosine and anidulafungin.

Clinical Isolates	MIC for 5-FC	MBEC for 5-FC	Variation (MBEC/MIC)	MIC for AND	MBEC for AND	Variation (MBEC/MIC)
***Ch***						
LIP*Ch*15	0.5 (S)	2 (S)	4×	0.25 (S)	2 (S)	8×
LIP*Ch*16	0.5 (S)	>64 (R)	>128×	0.125 (S)	>64 (R)	>512×
LIP*Ch*19	0.25 (S)	0.25 (S)	-	0.25 (S)	0.5 (S)	2×
LIP*Ch*27	0.5 (S)	8 (I)	16×	0.125 (S)	>8 (R)	>64×
LIP*Ch*30	0.5 (S)	64 (R)	128×	0.125 (S)	0.25 (S)	2×
LIP*Ch*34	0.125 (S)	>64 (R)	>512×	0.06 (S)	2 (S)	32×
LIP*Ch*36	0.25 (S)	>64 (R)	>256×	0.125 (S)	>8 (R)	>64×
***Cd***						
LIP*Ch*13	>64 (R)	>64 (R)	-	2 (S)	>8 (R)	4×
LIP*Ch*22	0.5 (S)	0.5 (S)	-	0.125 (S)	0.25 (S)	2×
LIP*Ch*23	0.25 (S)	1 (S)	4×	0.06 (S)	0.06 (S)	-
LIP*Ch*26	1 (S)	>64 (R)	>64×	0.06 (S)	4 (R)	64×
LIP*Ch*28	0.25 (S)	0.5 (S)	2×	0.06 (S)	0.06 (S)	-
LIP*Ch*29	0.25 (S)	2 (S)	8×	0.06 (S)	0.25 (S)	4×
LIP*Ch*31	1 (S)	1 (S)	-	0.06 (S)	0.125 (S)	2×
LIP*Ch*32	0.125 (S)	0.5 (S)	4×	0.06 (S)	0.125 (S)	2×
LIP*Ch*33	0.125 (S)	1 (S)	8×	0.06 (S)	0.125 (S)	2×
LIP*Ch*35	0.5 (S)	1 (S)	2×	0.06 (S)	0.125 (S)	2×
LIP*Ch*38	>64 (R)	>64 (R)	-	0.125 (S)	>8 (R)	>64×
***Chv***						
LIP*Ch*14	0.25 (S)	4 (S)	16×	0.125 (S)	0.125 (S)	-
LIP*Ch*18	0.25 (S)	8 (I)	32×	0.125 (S)	0.25 (S)	2×
LIP*Ch*20	0.25 (S)	8 (I)	32×	0.25 (S)	1 (S)	4×
LIP*Ch*21	0.25 (S)	4 (S)	16×	0.06 (S)	0.5 (S)	8×
LIP*Ch*24	0.25 (S)	32 (R)	128×	0.06 (S)	1 (S)	16×
LIP*Ch*25	0.25 (S)	8 (I)	32×	0.06 (S)	1 (S)	16×
LIP*Ch*37	<0.125 (S)	4 (S)	32×	0.06 (S)	0.25 (S)	4×

MIC, minimal inhibitory concentration of planktonic cells (mg/L); MBEC, minimal biofilm eradication concentration (mg/L); *Ch*, *C. haemulonii*; *Cd*, *C. duobushaemulonii*; *Chv*, *C. haemulonii* var. *vulnera*; 5-FC, flucytosine; AND, anidulafungin; S, susceptible; I, intermediate; R, resistant.

## Data Availability

Not applicable.
